# Factors associated with the level of self-management in elderly patients with chronic diseases: a pathway analysis

**DOI:** 10.1186/s12877-024-04956-9

**Published:** 2024-04-26

**Authors:** Zhiyang Cong, Mingshu Huo, Xing Jiang, Hongyu Yu

**Affiliations:** School of Nursing, Jinzhou Medical University, No.40, Section 3, Songpo Road, Linghe District, Jinzhou City, Liaoning Province P.R. China

**Keywords:** Chronic illness, Psychological capital, Family functioning, Sources of meaning in life, Self-management

## Abstract

**Background:**

To analyze the effects and pathways of factors such as psychological capital, family functioning, and sources of meaning in life on the level of self-management in elderly patients with chronic diseases and to provide a basis for the development of relevant nursing interventions in the future.

**Methods:**

Convenience sampling was used to select elderly patients with chronic diseases who underwent medical checkups and consultations at three community hospitals in Jinzhou city from March 2023 to October 2023, and the self-designed General Information Questionnaire (GIS), Psychological Capital of the Elderly Scale (PCE), Family Functioning Index Questionnaire (APGAR), Sources of Meaning of Life Scale for Older Adults(SMSE), and Self-Management Behavior of Chronic Patients Scale (SMCS) were used. SPSS 26.0 was used for data entry, one-way analysis, Pearson correlation analysis, and multiple linear regression were used to analyze the data, and Amos 17.0 was used to construct the structural equation model.

**Results:**

A total of 355 elderly patients with chronic diseases were included, and their self-management score was 74.75 ± 12.93, which was moderate. The results of the influencing factor analysis showed that the influencing factors of the self-management level of elderly chronic disease patients were age, years of illness, psychological capital, family functioning, and sources of meaning in life (*p* < 0.05). Path analysis revealed that sources of meaning in life were a partial mediator of the relationship between psychological capital and self-management, with an effect value of 0.166 (95% CI: 0.042,0.391), accounting for 37.6% of the total effect; life meaning was a partial mediator of family functioning and self-management level, with an effect value of 0.231 (95% CI: 0.040,0.452), accounting for 54.0% of the total effect. accounting for 54.0% of the total effect.

**Conclusion:**

The self-management of elderly patients with chronic diseases is intermediate. Healthcare professionals should actively implement holistic healthcare management measures from the family aspect to help patients understand the meaning of life and improve the level of patients’ psychological capital to improve the self-management level of elderly patients with chronic diseases.

## Background

According to data released by the World Health Organization (WHO) [1], chronic diseases account for six of the top ten causes of death worldwide. However, there is a lack of existing research on chronic diseases as a whole, with most studies focusing on specific chronic diseases, such as high blood pressure or heart disease [2]. In some developed countries, especially Australia [3, 4], the Netherlands [5] Sweden [6, 7] and Canada [8, 9], pioneering work has been done on chronic diseases. These studies have shown that as older people’s body functions decline, their lack of awareness of chronic diseases [10] and inadequate self-management skills [11], and other reasons, it is receiving increasing attention from the international community [12]. According to official statistics, in 2019, China’s population aged 60 years and older reached 254 million. By 2040, this number is expected to increase to 402 million, accounting for approximately 28% of the population [13]. Nearly 80% of deaths among people aged 60 and over are caused by chronic diseases [14, 15]. This has far-reaching implications for health in China. Some studies have noted that to manage chronic diseases properly, long-term health management rather than intermittent treatment should be provided to the aging population and that care for elderly people should be primarily community- and home-based rather than hospital-based [13]. The World Health Organization (WHO) has also noted that chronic disease management should emphasize empowering individuals and communities and that promoting patient self-management behaviors is more effective than any intervention [16].

Understanding the factors that influence self-management in elderly patients with chronic diseases is fundamental to designing effective interventions to optimize self-management, and by combing through the existing literature, most studies investigating the factors that influence self-management behaviour lack a theoretical framework [17]. Inconsistencies in the effectiveness of existing interventions to improve self-management in older people with chronic conditions can be partly explained by the fact that self-management interventions often lack the theoretical models that underpin intervention development [18]. For example, a review of the promotion of self-management in people with chronic conditions concluded that very few studies used explicitly theory-based interventions, and Michie et al. also emphasized that the design of interventions rarely applies underlying theoretical models [19].

**Fig. 1:**
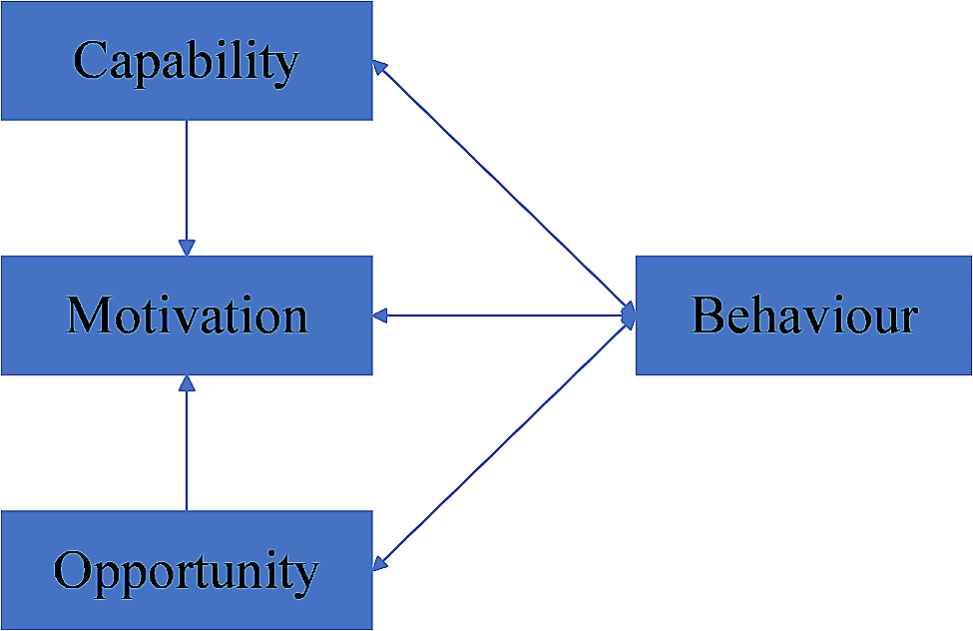
COM-B model

The Capability, Opportunity, and Motivation - Behaviour (COM-B) model, first proposed by Michie et al. [19] in 2011, suggests that the occurrence of behaviour consists of three required components: ability, opportunity and motivation, as illustrated in Fig. 1. The COM-B model is the only behaviour change model [19] that links behavioural influences to interventions and can be used to develop effective, theory-based interventions. In this model, capabilities (psychological or physiological capacities, such as self-efficacy and hope), opportunities (physical and social environments, such as family influences), and motives (reflective and autonomous mechanisms, such as goals and motivation) interact to produce behaviour [18]. Motivation mediates between ability, opportunity, and behaviour. Based on the COM-B model and previous research, we used competence as psychological capital, opportunity as a family function, life meaning sources as a motivator for self-management, and behaviour as self-management.

Luthans [20] defines psychological capital as a positive mental state covering four main elements: self-efficacy, hope, optimism and resilience. Some scholars consider it to be a positive state of mind or mental ability of an individual in the appropriate situation. Self-efficacy is a recognized mediator in self-management programs [21]. Interventions can enhance patients’ self-efficacy, lead to positive changes in health behaviours [22–24], and improve health outcomes [25–28]. However, it is not clear whether this reflects a direct link between psychological capital and self-management.

At the opportunity level, family support from family members and friends provides emotional and material security to patients, relieving them of financial or psychological burdens and enabling them to better manage their lives [29]. Given that the majority of a person’s daily disease management is spent at home, it is unsurprising that home function is integral to improving patient self-management.

A life meaning source is the source of an individual’s source of meaning in life and corresponds to the goals and motivations for each stage of life [30, 31]. Research has shown that the meaning of life is closely linked to self and others, that family relationships are the main sources of the meaning of life for older people, and that the meaning of life has a significant impact on the quality of life, life satisfaction, and interpersonal relationships of older people, which can contribute to healthy aging [32]. Yet little is known about the mediating role of life-meaning sources in the relationship between other possible factors (e.g., psychological capital) and self-management in patients with chronic illness.

**Fig. 2:**
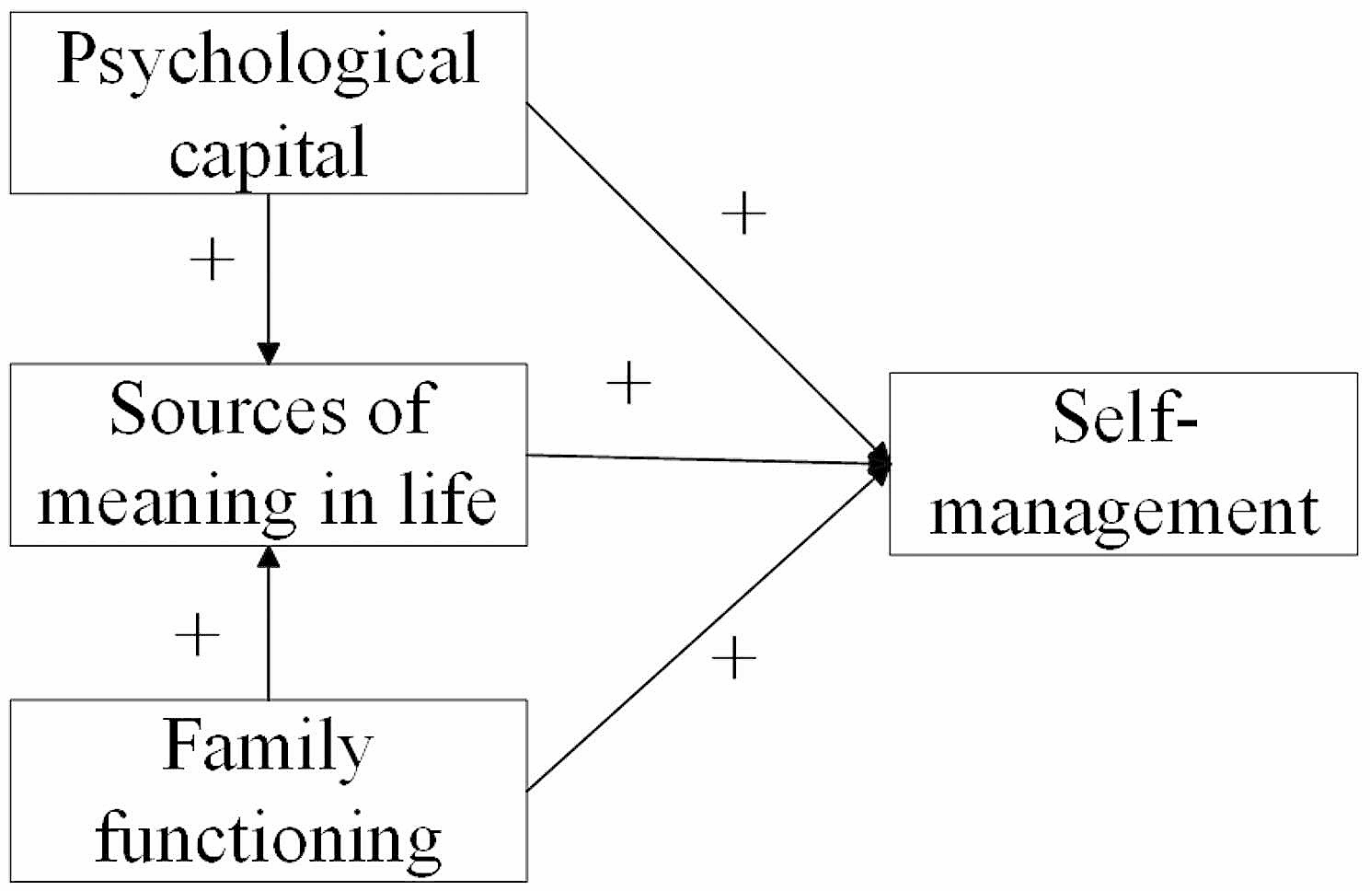
Hypothetical model

Therefore, in the elderly chronic disease population, we hypothesized that H1a: psychological capital is positively related to self-management; H1b: psychological capital is positively related to sources of meaning in life; H1c: sources of meaning in life are positively related to self-management; H2a: family functioning is positively related to self-management; H2b: family functioning is positively related to sources of meaning in life; H3a: Sources of meaning in life mediate between psychological capital and self-management; H3b: Sources of meaning in life mediate between family functioning and self-management. The hypothesized model of this study is shown in Fig. 2.

## Method

### Study design and participants

The convenience sampling method was used to select one of the three community health service centers in Jinzhou city as the location of the investigation, and elderly chronic patients who visited the centers for medical check-ups and consultations were selected as the subjects of the survey. The inclusion criteria for patients were as follows: 1) ≥ 60 years of age; 2) met the diagnostic criteria for chronic disease and had been diagnosed by a hospital at the second level or above; 3) were able to communicate effectively and complete the questionnaire independently or with the assistance of others by their own wishes; and 4) provided informed consent and participated voluntarily. Exclusion criteria are refusal to investigate, inability to complete the investigation, or lack of cooperation with the investigation.

The study’s questionnaire contained 26 variables. According to Kendall’s rough estimation method for sample size: sample size = [number of variables × (5–10) × (1 + (10-15%)] [33], the sample size should be 5–10 times the number of variables, with a range of 130–260 cases. Considering the 20% sample attrition rate and potential errors due to convenience sampling, the estimated sample size was adjusted to 156–310 cases. A sample size of 200 or more was preferred given structural equation modelling [34]. To ensure the reliability of the results, 380 questionnaires were distributed, with 355 valid responses and a validity rate of 92.21%.

#### Ethical approval

for this study was obtained from the Ethics Committee of Jinzhou Medical University (JZMULL2023011). The questionnaire method was used to collect the data. Master of nursing who had undergone relevant training and were familiar with the purpose of the study were administered with the consent of the relevant department of the CHC. The purpose and content of the survey were explained to the respondents by the researcher, who promised anonymity and confidentiality. Respondents filled out an informed consent form and completed a questionnaire, which was collected on the spot by the researcher on the same day (respondents were in a separate room from the researcher to protect patient privacy). If the respondents could not read and write, the researcher read the questions one by one, and the subjects answered them one by one. The researcher subsequently filled in the questionnaire on their behalf, and the process of completing the questionnaire ensured that the data were true and accurate. Finally, the researcher excluded invalid questionnaires, entered valid data in pairs, and randomly reviewed 20% of the data to ensure that the data were true and accurate.

### Measures

#### General information questionnaire (GIS)

The scale was developed by the researcher based on the study objectives and group characteristics after the researcher reviewed relevant information and developed the scale on his own. The scale has six entries, including gender, age, education level, number of chronic diseases, number of years of illness, and number of hospitalizations due to chronic diseases.

#### Psychological capital scale for older adults(PCE)

The research tool used was the Shi [35]. The Psychological Capital Scale for the Elderly, compiled by Shi Hui, adopts a 5-point Likert scale with a total possible score of 20–100 points and consists of 4 dimensions, namely, self-actualization, perseverance, honesty and steadfastness, and thankfulness and dedication, for a total of 20 entries. The scale is grouped according to the quartile method according to different levels of psychological capital, with ≥ 72 indicating a low level, 73 ∼ 79 indicating a medium level, and ≥ 80 indicating a high level; the Cronbach’s α of the scale is 0.865.

#### Family functioning index questionnaire (APGAR)

The scale was developed by Smilkstei [36] and comprises 5 dimensions, i.e., cooperation, adaptation, intimacy, growth, and emotion. A 3-point scoring method is used to divide the total score into three levels corresponding to different statuses of family functioning. A score ranging from 7 to 10 points indicates that family functioning is at a high level and functioning status is good; 4 to 6 points indicate that family functioning is at a low level and there is a current state of moderate impairment; and 0 to 3 points indicate that family functioning is currently at a low level and there is a serious obstacle. The results of many related surveys at home and abroad show that the Cronbach’s alpha coefficient is 0.813–0.857, indicating high reliability and validity.

#### Sources of meaning of life scale for older adults (SMSE)

The research instrument was developed by Jingjing Zhou [37] and contains 28 items, namely, family, sense of value, social support, leisure activities, personal development, and life security, with 6 dimensions. The total score of this scale ranges from 28 to 196 points and is grouped by quartile. Different levels of life meaning sources are assigned. A score ≤ 148 indicates a low level of life meaning, 149 ∼ 159 indicates a medium level of life meaning, and ≥ 160 indicates a high level of life meaning. The Cronbach’s alpha coefficient of the scale was 0.924.

#### Chronic patient self-management behavior scale(SMCS)

The scale was developed by Qiao Han [38] and compiled by Qiao Han. The scale consists of 21 items in 5 dimensions: diet, exercise, medication, emotion, family and society. A 5-point Likert scale was used, with a total score ranging from 0 to 100 points indicating different levels of self-management; 20–60 points indicate that self-management is currently at a low level, 60–80 points indicate that self-management is at a medium level, and 80–100 points indicate that self-management is currently at a high level. The Cronbach’s alpha coefficient of the scale is 0.957.

### Statistical analysis

Data were analyzed using SPSS 20.0 and Amos 17.0 software; general information was analyzed by descriptive statistics; count data were described by frequency and percentage, and measurement data were described by mean ± standard deviation; the correlation between self-management and each variable was analyzed by Pearson’s correlation analysis; the total score of self-management behavioural scale was used as the dependent variable, and the total score of psychological capital scale of elderly people, the family functioning index questionnaire, and the sources of meaning of life scale of elderly people were used as the independent variables. Questionnaire, and Sources of Meaning in Older Adults’ Lives Scale as the dependent variables, and multivariate linear regression analyses were used. Amos 17.0 software was used for path analysis. The model is valid when the fit indices GFI, CFI, NFI, TLI and IFI > 0.9 and RMSEA < 0.8 [39]. *p* < 0.05 was considered a statistically significant difference. The mediating role of life meaning sources was verified using the Bootstrap program.

## Results

### Participants’ characteristics

A total of 355 elderly patients with chronic diseases aged 74.75 ± 12.93 were included in this study, and the remaining data are shown in Table 1. The results of univariate analysis showed that the difference in self-management level scores was statistically significant (*p* < 0.05) when comparing patients of different ages and years of chronic disease diagnosis, as shown in Table 1.

**Table 1:** Demographic and disease characteristics of participants (*n* = 355)

Characteristics	N (%)	Mean ± SD	T/F	*p*
Gender			0.033	0.856
male	200 (56.3)	74.86 ± 12.42		
Female	155 (43.7)	74.61 ± 13.59		
Age (years)			6.922	0.001
60~	272 (76.6)	75.94 ± 11.86		
70~	78 (22.0)	70.19 ± 15.59		
80~	5 (1.4)	81.60 ± 3.78		
educational attainment			0.918	0.432
Primary and below	46 (13)	76.96 ± 12.04		
junior high school	85 (23.9)	73.24 ± 13.29		
High school	102 (28.7)	74.43 ± 13.96		
College and above	122 (34.4)	75.25 ± 12.07		
Number of chronic diseases			0.665	0.574
1	297 (83.7)	74.82 ± 12.44		
2	38 (10.7)	74.13 ± 14.61		
3	18 (5.1)	76.28 ± 14.68		
≥ 4	2 (0.6)	63.00 ± 38.18		
Years of chronic disease diagnosis			4.749	0.003
≤ 3	193 (54.4)	73.36 ± 13.55		
3~	37 (10.4)	74.16 ± 14.37		
6~	105 (29.6)	78.46 ± 9.20		
10~	20 (5.6)	69.85 ± 16.78		
Number of hospitalizations for chronic diseases in the last year			0.529	0.590
0	297 (83.7)	74.47 ± 13.21		
1	31 (8.7)	75.50 ± 13.43		
≥ 2	27 (7.6)	76.96 ± 8.79		

SD: standard deviation; T: T-value; F: F-value

### Statistical description and Pearson correlation analysis of variables

The total psychological capital score of the elderly patients with chronic diseases was 73.01 ± 11.09, the score was at the lower middle level, the total score for family functioning was 7.55 ± 2.45, the score was at the lower high level, the total score for sources of meaning in life was 126.21 ± 22.10, the score was at the low level, and the total score for self-management was 74.75 ± 12.93 (Table 2). According to the results of Pearson’s correlation analysis, psychological capital is positively correlated with family functioning, sources of meaning in life, and level of self-management.

**Table 2:** Means, standard deviations, and Pearson correlations of the variables used in this study

variant	Mean ± SD	1	2	3	4
1. psychological capital	73.01 ± 11.09	1			
2. family function	7.55 ± 2.45	0.398^**^	1		
3. sources of meaning for life	126.21 ± 22.10	0.487^**^	0.589^**^	1	
4. self-management	74.75 ± 12.93	0.489^**^	0.472^**^	0.491^**^	1

SD: standard deviation; **: *p* < 0.01

### Analysis of multiple linear regression

Three multiple linear regression analyses were conducted with psychological capital, sources of meaning in life and family functioning as the main independent variables, demographic characteristics as control variables and self-management as the dependent variable. The results showed Table 3 that according to model 1, general information explained 1.20% of the standardized variance (F = 3.147,*p* < 0.01); the multiple linear regression analysis of model 2 showed that psychological capital and family functioning significantly and positively predicted self-management, explaining 33.10% of the standardized variance (F = 44.818, *p* < 0.01); and model 3, based on model 2 added the meaning of life sources, positively predicted self-management, explaining 35.70% of the standardized variance (F = 40.290, *p* < 0.01).

**Table 3:** Regression analyses with self-management as dependent variables

Outcome variable	Model1			Model2			Model3		
	β	*p*	95%CI	β	*p*	95% CI	β	*p*	95% CI
(a person’s) age	-0.089	0.000	69.742,104.215	-0.048	0.055	-0.4, 0.004	-0.087	0.043	-0.421, 0.007
Years of chronic disease diagnosis	0.102	0.094	-0.475, 0. 037	0.032	0.468	-0.6, 1.494	0.045	0.301	-0.508, 1.638
psychological capital	-	-	-	0.349	0.000	0.29, 0.516	0.281	0.000	0.213, 0.443
family function	-	-	-	0.334	0.000	1.27, 2.256	0.232	0.000	0.670, 1.780
Sources of meaning for life	-	-	-	-	-	-	0.218	0.000	0.03, 0.192
F	3.147			44.818			40.290		
R^2^	0.018			0.339			0.266		
Adjusted R^2^	0.012			0.331			0.357		

β: Standardized regression coefficients; 95% CI:95%confidence interval

### Path analysis

**Fig. 3:**
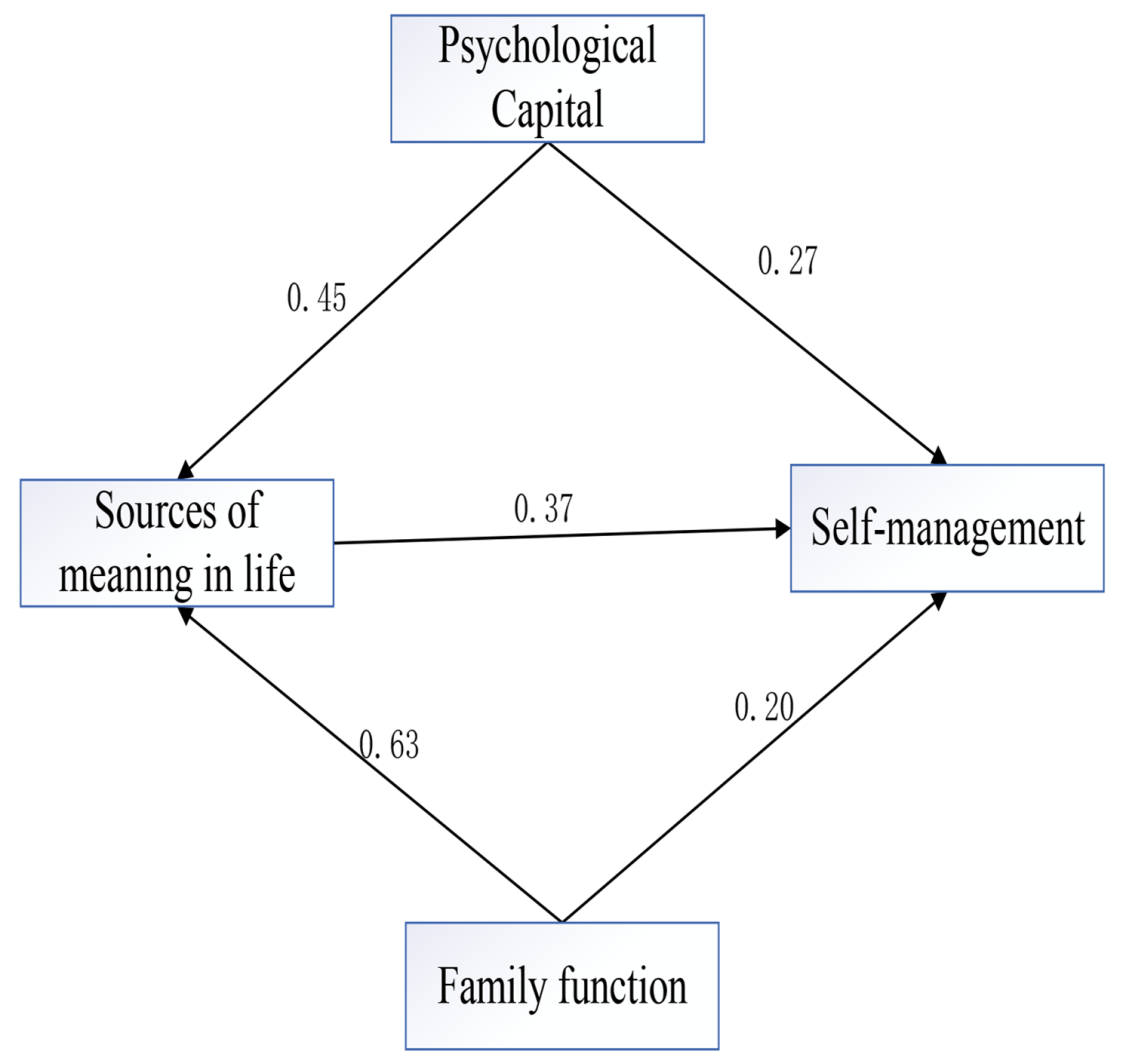
Result of the path model and path coefficients

The chained mediation model was constructed with psychological capital and family functioning as the independent variables, the sources of meaning in life as the mediating variable, and the level of self-management as the dependent variable. The results showed goodness-of-fit index (GFI) = 0.929, comparative fit index (CFI) = 0.926, normative fit index (NFI) = 0.879, nonnormative fit index (TLI) = 0.911, incremental fit index (IFI) = 0.927, and root mean square of the error of approximation (RMSEA) = 0.061. The various fit indicators are excellent, and the model is acceptable. The mediating role-fitting model is shown in Fig. 3.

The bootstrap method was used to randomly select 5,000 data points (*n* = 355) for the mediation effect test. The results showed that psychological capital has a direct effect on the level of self-management (95% CI: 0.003,0.543), family functioning has a direct effect on the level of self-management (95% CI: 0.001,0.369), the value of the direct effect is 0.275, 0.198, the indirect effect of patients’ psychological capital from life meaning sources to self-management (95% CI: 0.042,0.391), and patients’ family functioning from life meaning sources to self-management (95% CI: 0.040,0.452) was significant, with mediating effect values of 0.166 and 0.231 (*p* < 0.001). The family functioning to self-management mediating effect was 53.97% and the psychological capital to self-management mediating effect was 37.64%. Bias-corrected upper and lower intervals without passing through 0 is the criterion for proving the validity of direct and indirect effects. Thus, the meaning of life sources plays a partial mediating role in the relationship between psychological capital, family functioning and self-management in elderly patients with chronic illness. The results of structural model evaluation and hypothesis testing are shown in Table 4, all hypotheses are statistically significant.

**Table 4:** Results of mediation effect tests between psychological capital, family functioning, sources of meaning in life and self-management, and hypothesis testing

	Path	Estimate	S.E.	Bias-corrected 95%CI	Effect Ratio (%)	Results
	Indirect Effect					
H3a	X→M→Y	0.166	0.085	0.042,0.391	37.64	Accepted
H3b	N→M→Y	0.231	0.105	0.040,0.452	53.97	Accepted
	Direct Effect					
H1b	X→M	0.451	0.095	0.264,0.635	-	Accepted
H2b	N→M	0.628	0.056	0.512,0.731	-	Accepted
H1a	X→Y	0.275	0.138	0.003,0.543	62.36	Accepted
H2a	N→Y	0.198	0.095	0.001,0.369	46.26	Accepted
H1c	M→Y	0.367	0.138	0.054,0.683	-	
	Total Effect					
H1a	X→Y	0.441	0.092	0.260,0.614	100	Accepted
H2a	N→Y	0.428	0.057	0.310,0.535	100	Accepted

Note: X is psychological capital; M is Sources of meaning in life; N is Family functioning; Y is Self-management

## Discussion

The results of this study showed that the self-management level of middle-aged and elderly patients with chronic diseases was 74.75 ± 12.93, which was moderate and slightly lower than the findings of Zhang et al. [40]. The reason for this difference may be that the sample in this study was small, and Zhang’s study population was hypertensive elderly people who had more healthcare resources and were generally more concerned about their health. Second, the difference in the results was due to the different research tools used. Whereas Dixon et al.’ s [41] findings suggest that it appears that most people with chronic illnesses understand the importance of self-management, yet the challenging nature of achieving self-management (e.g. feeling that medication doesn’t do anything, and lack of trust in advice given by professionals) can prevent people from doing it effectively. Research on self-management has focused primarily on diagnosis-specific conditions, such as arthritis [42], diabetes [43, 44], or chronic kidney failure [45], a focus that makes it difficult to distinguish between diagnosis-specific problems and those that apply to patients with chronic conditions in general.

Psychological capital, as a positive psychological resource possessed by individuals, sets the conditions for self-management integration [46] and is positively related to self-management levels. In general, behaviour depends on this psychological resource, which is one of the most obvious features of healthy adult behaviour [47]. This result is similar to Mcdonald-Miszczak’s study on self-care behaviours in heart disease and hypertension [48], where self-efficacy and overall sense of worth were better predictors of self-management behaviours in patients with heart disease and hypertension. Under the influence of positive psychological capital, patients can demonstrate character strengths, increase positive optimism, and establish a defense mechanism that can withstand the distress of their chronic diseases [49] thus improving self-management. Psychologists believe that when individuals have healthy psychological capital, they are better able to see the positive aspects of their lives and actively seek solutions to their problems, which makes it easier for them to acquire the expertise to accomplish self-management of their illnesses [50].

The findings of Epstein et al. [51] and Bennich et al. [52] research are consistent with the results of the present study that there is a positive correlation between family functioning and self-management. Several studies have also highlighted the importance of promoting this resource in hypertensive and diabetic populations [53–55], with family support playing a crucial role in old age as individuals age and experience more stressful life events (e.g., the onset of chronic disease) [56, 57]. In this study, it can be understood that self-management behaviours of elderly patients with chronic illnesses tend to occur in the important environment of the family, and a well-functioning family can provide positive emotional support for elderly patients with chronic illnesses, so that the elderly show beneficial effects in terms of happiness and depression [58], stabilize the neuroendocrine system and increasing healthy behaviours [59].

Psychological capital positively predicts the sources of meaning in life. Fewer studies have been found on psychological capital and sense of meaning in life [60–62]. Our findings are similar to Jonsén’s [63]. Patients with high levels of psychological capital can maintain patients’ hope and optimism in dealing with their illnesses and will find beauty, joy, and happiness in good living environments, which is beneficial for eliminating negative emotions such as loneliness and depression among elderly patients with chronic illnesses and helping to build a positive psychological state in elderly people, who in turn can experience a more profound sources of meaning in life.

Family functioning is positively correlated with the sources of meaning of life in older chronically ill patients, consistent with the findings of previous studies [64, 65]. Families can increase the amount of material or information provided to give people a sense of joy and meaning, especially for older people who need more care and help [59]. Cheng et al. conducted a cross-sectional survey in China and found that the psychological status of family functioning was significantly correlated in both urban and rural areas [66]. Zou et al. [67] also found that dysfunctional family dynamics may contribute to the onset of poor mental status when older adults are ill and hospitalized. Elderly patients with chronic diseases can obtain joy from a harmonious and happy family atmosphere, experience responsibility and affection, and feel the beauty of life, thus constructing a consciousness of caring for life and perceiving the importance of life, which is highly important for enhancing their sources of meaning in life.

Patient sources of meaning in life positively predict the level of self-management, which is consistent with the findings of Zhou [68]. An individual’s internal negotiation between self-management and life goals is one of the key factors in self-management [46]. Kralik et al. describe the transition to self-management as a process by which people with chronic illnesses achieve a sense of balance in the pursuit of a meaningful life as they go through their illness and its treatment [42]. Whittemore et al. present similar findings about self-management in diabetics; they describe how interpersonal conflict often creates barriers to maintaining dietary and exercise behaviours [44]. The individual’s internal pursuit of life goals is critical to integrating self-management [46].

The results of this study are consistent with our initial hypothesis. Life meaning sources mediate the relationship between family functioning, psychological capital, and self-management of chronically ill patients, in line with the COM-B model in which capability (psychological capital), and opportunity (family functioning) influence behaviour through the motivational dimension (life meaning sources).

Similar findings, such as sources of meaning in life, and family functioning being related to self-management, have been reported in previous studies [69, 70], but the pathways mechanisms by which psychological capital and family functioning may influence self-management are not yet well understood. One study describes [71, 72] self-management as a chronological process in which individuals experience a turnaround from not knowing much about illness and self-management (e.g., a sudden epiphany about the meaning of life), after which they decide to self-manage and incorporate self-management practices into their daily lives [46]. This process is complex and includes a motivated individual who, as the process progresses, changes personal values goals, and motivations, and thus changes lifestyle and self-management behaviours [43, 44]. The mediating role of life meaning sources was demonstrated in this study, where higher psychological capital and family functioning can enhance goals and motivation to overcome chronic disease, promote the practice of healthy lifestyle behaviours, and improve self-management [56, 73], and even reduce the risk of illness and death [56, 74]. Ingadottir et al. had similar findings in their study of diabetic patients, where positive minded diabetic patients control their cravings, thereby increasing their sense of self-meaning and motivation in the disease management process and working to change their behaviours towards dietary restriction [43, 44]. Although there is no more conclusive evidence of a mediating effect, these results support our finding that the sources of meaning in life at least partially explain the relationship between family functioning, psychological capital, and self-management in older chronically ill patients. This finding highlights the importance of developing psychological family intervention programs.

Several limitations of this study should be addressed. Firstly, this study is a cross-sectional investigation and the relationship between these variables cannot be considered causal. Second, the validity of the research instrument is uncertain, and it is recommended that the research instrument be improved or modified in future experiments to increase the accuracy of the measurements. Thirdly, the participants in our study were from a single city in China and therefore may not be representative of all Chinese patients with chronic diseases; therefore, it is recommended that the scope of data collection be expanded in future studies.

## Conclusions

The self-management of elderly patients with chronic diseases is at an intermediate level, with age, years of illness, psychological capital, family functioning, and sources of meaning of life as influencing factors, and the sources of meaning of life have a mediating role between psychological capital, family functioning, and self-management. Therefore, healthcare professionals should actively promote holistic health measures for family members to effectively improve their self-management. Given that this was a cross-sectional study with a limited number of respondents, additional large-sample, multicenter longitudinal studies could be conducted to investigate self-management and its trajectory of change in elderly patients with chronic diseases.

## Data Availability

Upon a reasonable request, the corresponding author will make the datasets used in this study public.
